# Neuronal adenosine A_2A_ receptors signal ergogenic effects of caffeine

**DOI:** 10.1038/s41598-020-69660-1

**Published:** 2020-08-07

**Authors:** Aderbal S. Aguiar, Ana Elisa Speck, Paula M. Canas, Rodrigo A. Cunha

**Affiliations:** 1 CNC-Center for Neuroscience and Cell Biology, University of Coimbra, 3004-504 Coimbra, Portugal; 2 Biology of Exercise Lab, Department of Health Sciences, UFSC-Federal University of Santa Catarina, Araranguá, SC 88905-120 Brazil; 3 FMUC – Faculty of Medicine, University of Coimbra, 3004-504 Coimbra, Portugal

**Keywords:** Metabolism, Neurophysiology

## Abstract

Caffeine is one of the most used ergogenic aid for physical exercise and sports. However, its mechanism of action is still controversial. The adenosinergic hypothesis is promising due to the pharmacology of caffeine, a nonselective antagonist of adenosine A_1_ and A_2A_ receptors. We now investigated A_2A_R as a possible ergogenic mechanism through pharmacological and genetic inactivation. Forty-two adult females (20.0 ± 0.2 g) and 40 male mice (23.9 ± 0.4 g) from a global and forebrain A_2A_R knockout (KO) colony ran an incremental exercise test with indirect calorimetry (V̇O_2_ and RER). We administered caffeine (15 mg/kg, i.p., nonselective) and SCH 58261 (1 mg/kg, i.p., selective A_2A_R antagonist) 15 min before the open field and exercise tests. We also evaluated the estrous cycle and infrared temperature immediately at the end of the exercise test. Caffeine and SCH 58621 were psychostimulant. Moreover, Caffeine and SCH 58621 were ergogenic, that is, they increased V̇O_2_max, running power, and critical power, showing that A_2A_R antagonism is ergogenic. Furthermore, the ergogenic effects of caffeine were abrogated in global and forebrain A_2A_R KO mice, showing that the antagonism of A_2A_R in forebrain neurons is responsible for the ergogenic action of caffeine. Furthermore, caffeine modified the exercising metabolism in an A_2A_R-dependent manner, and A_2A_R was paramount for exercise thermoregulation.

## Introduction

The natural plant alkaloid caffeine (1,3,7-trimethylxantine) is one of the most common ergogenic substances for physical activity practitioners and athletes^1–10^. Caffeine increases endurance^1,8–12^, intermittent^7,13,14^ and resistance^4,15^ exercise in humans. In rodents, its ergogenic effects are conserved because caffeine increases running time on the treadmill at constant^16,17^ and accelerated speeds^18,19^. Sports sciences promote nonselective phosphodiesterase (PDE) inhibition^7,8^ and increased calcium mobilization^2,7,8^ as mechanisms for these ergogenic effects. However, the primary pharmacological effect of caffeine is the nonselective antagonism of adenosine A_1_ and A_2A_ receptors (A_1_R, A_2A_R)^20–23^.

Adenosine can act as an inhibitory modulator of the Central Nervous System (CNS) associated with tiredness and drowsiness^24–29^. During exercise, circulating ADP/AMP/adenosine levels increase due to ATP hydrolysis^30,31^. However, there is still no substantial evidence on the role of adenosine in exercise-induced fatigue. It is just known that the nonselective A_1_R and A_2A_R agonist 5′-(N-ethylcarboxamido)adenosine (NECA), injected into the rat brain, abolishes the ergogenic effects of caffeine^16^.

Since there is increasing evidence that the adenosine modulation system critically controls allostasis^29^ and A_2A_R have a crucial role in the ability of caffeine to normalize brain function^30^^,^ we hypothesized that caffeine decreases fatigue during exercise through antagonism of A_2A_R in the CNS. We combined the use of pharmacology (SCH 58261 and caffeine) and transgenic mice with tissue-selective deletion of A_2A_R, to test this hypothesis in an incremental running test with indirect calorimetry (or ergospirometry). A_2A_R knockout (KO) mice allow assessing if the ergogenic effect of caffeine persists in the absence of A_2A_R; the use of SCH 58261, the current reference for A_2A_R antagonists^32,33^, allows directly assessing the ergogenic role of A_2A_R. SCH 58261 has excellent selectivity and affinity for A_2A_R^32,33^, and affords motor benefits in animal models of Parkinson's disease as does caffeine, supporting the recent FDA approval of the A_2A_R antagonist Istradefylline for PD treatment^33^. Our goal is to assess the ergogenicity of A_2A_R using the pharmacological and genetic tools described above.

## Methods

### Animals and A_2A_R KO colony

We used 40 male (23.9 ± 0.4 g, 8–10 weeks old) and 42 female mice (20.0 ± 0.2 g, 8–10 weeks old) from our global-A_2A_R (A_2A_R KO) and forebrain-A_2A_R KO (fb-A_2A_R KO) inbred colony^34,35^ and wild type littermates. The sample size for ANOVA comparison had α = 0.05 and β = 0.8.

The inactivation of exon 2 of A_2A_R in a near congenic (N6) C57BL/6 genetic background was the method of generating A_2A_R KO mice^36,37^. We also have good experience with treadmill running in this strain^38–40^. A_2A_R KO mice and wild type littermates were matched for sex and age for each experiment. The Cre-loxP strategy, crossing floxed A_2A_R mice with mice expressing CRE under the forebrain-selective promoter CAM-kinase 2, allows generating fb-A_2A_R KO mice, as previously described^34,41^. We used global A_2A_R KO females and fb-A_2A_R KO males due to the characteristic of our colony.

Mice were housed in collective cages in HEPA-filtered ventilated racks (n = 3–5) under a controlled environment (12 h light–dark cycle, lights on at 7 AM, and room temperature of 21 ± 1 °C) with ad libitum access to food and water. Housing and handling were performed according to European Union guidelines (2010/63). The Ethical Committee of the Center for Neuroscience and Cell Biology (University of Coimbra) approved the study.

### Drugs

7-(2-phenylethyl)-5-amino-2-(2-furyl)-pyrazolo-[4,3-e]-1,2,4-triazolo[1,5-c]pyrimidine (SCH 58261) was solubilized in 10% dimethyl sulfoxide (DMSO) in 0.9% NaCl – saline. Caffeine was dissolved in saline. SCH 58261 and caffeine were freshly prepared and administered intraperitoneally (volume of 10 mL/kg body mass). Caffeine, DMSO, and NaCl were obtained from Sigma-Aldrich and SCH 58261 from Tocris. The doses used of SCH 58261 (1 mg/kg) and caffeine (15 mg/kg) were based on our previous experience in the use of these compounds^42,43^ and pilot studies.

### Experimental design

Fig.S1 shows the experimental design. The habituation of handling, injections (0.9% NaCl, i.p.), and moving treadmill (15 cm/s) occurred in the first three days of the experiment. The animals were treated with SCH 58261 (1 mg/kg, i.p.) and caffeine (15 mg/kg, i.p.) on days 4 and 5, 15 min before testing in the open field (4th day) and ergospirometry (5th day). The experiments took place between 9 AM and 5 PM, within the light phase of the mouse dark/light cycle, in a sound-attenuated and temperature/humidity controlled room (20.3 ± 0.6 °C and 62.8 ± 0.4% H_2_O) under low-intensity light (≈ 10 lx). The open field apparatus and the treadmill were cleaned with 10% ethanol between individual experiments. The allocation for the experimental groups was random. For each test, the experimental unit was an individual animal.

### Open field

Mice explored an unaccustomed open field (38 × 38 cm) for 15 min. Locomotion was analyzed using an ANY-Maze video tracking system (Stoelting Co.).

### Ergospirometry

Mice were accustomed to a single-lane treadmill (Panlab LE8710, Harvard apparatus) at speed 15 cm/s (10 min, slope 5°, 0.2 mA) with a 24 h interval between each habituation session (Fig. S1). The incremental running protocol started at 15 cm/s, with an increment of 5 cm/s every 2 min at 5° inclination^40^. The exercise lasted until running exhaustion, defined by the inability of the animal to leave the electrical grid for 5 s^40,44^.

Oxygen uptake (V̇O_2_) and carbon dioxide production (V̇CO_2_) were estimated in a metabolic chamber (Gas Analyzer ML206, 23 × 5 × 5 cm, AD Instruments, Harvard) coupled to the treadmill. The animals remained in the chamber for 15 min before exercise testing. Atmospheric air (≈21% O_2_, ≈0.03% CO_2_) was renewed at a rate 120 mL/min, using the same sampling rate for the LASER oxygen sensor (Oxigraf X2004, resolution 0.01%) and infrared carbon dioxide sensor (Servomex Model 15050, resolution 0.1%).

We estimated the running and critical power output for a treadmill based on a standard conversion of the vertical work, body weight, and running speed^40,45,46^. Running power is the sum (Σ) of all stages of the exercise test, and critical power is the running work performed above V̇O_2_max.

### Vaginal cytology

We evaluated the estrous cycle immediately after the exercise test, through 4–5 consecutive vaginal lavages (with 40–50 μL of distilled H_2_O) then mounted on gelatinized slides (76 × 26 mm)^47,48^. These procedures lasted no more than 3–5 min, and there were no significant time delays between behavioral experiments and fluid collection for vaginal cytology.

The vaginal smear was desiccated at room temperature and covered with 0.1% crystal violet for 1 min, then twice washed with 1 mL H_2_O and desiccated at room temperature^47,48^. The slides were mounted with Eukitt medium (Sigma-Aldrich) and evaluated under an optical microscope at 1x, 5x, and 20x (Zeiss Axio Imager 2). We evaluated three cell types for determining the estrous cycle: nucleated epithelial cells, cornified epithelial cells, and leukocytes. Cellular prevalence defined proestrus (nucleated), estrus (cornified), metestrus (all types in the same proportion), and diestrus (leukocytes)^47,48^.

### Thermal imaging

**Figure 1:**
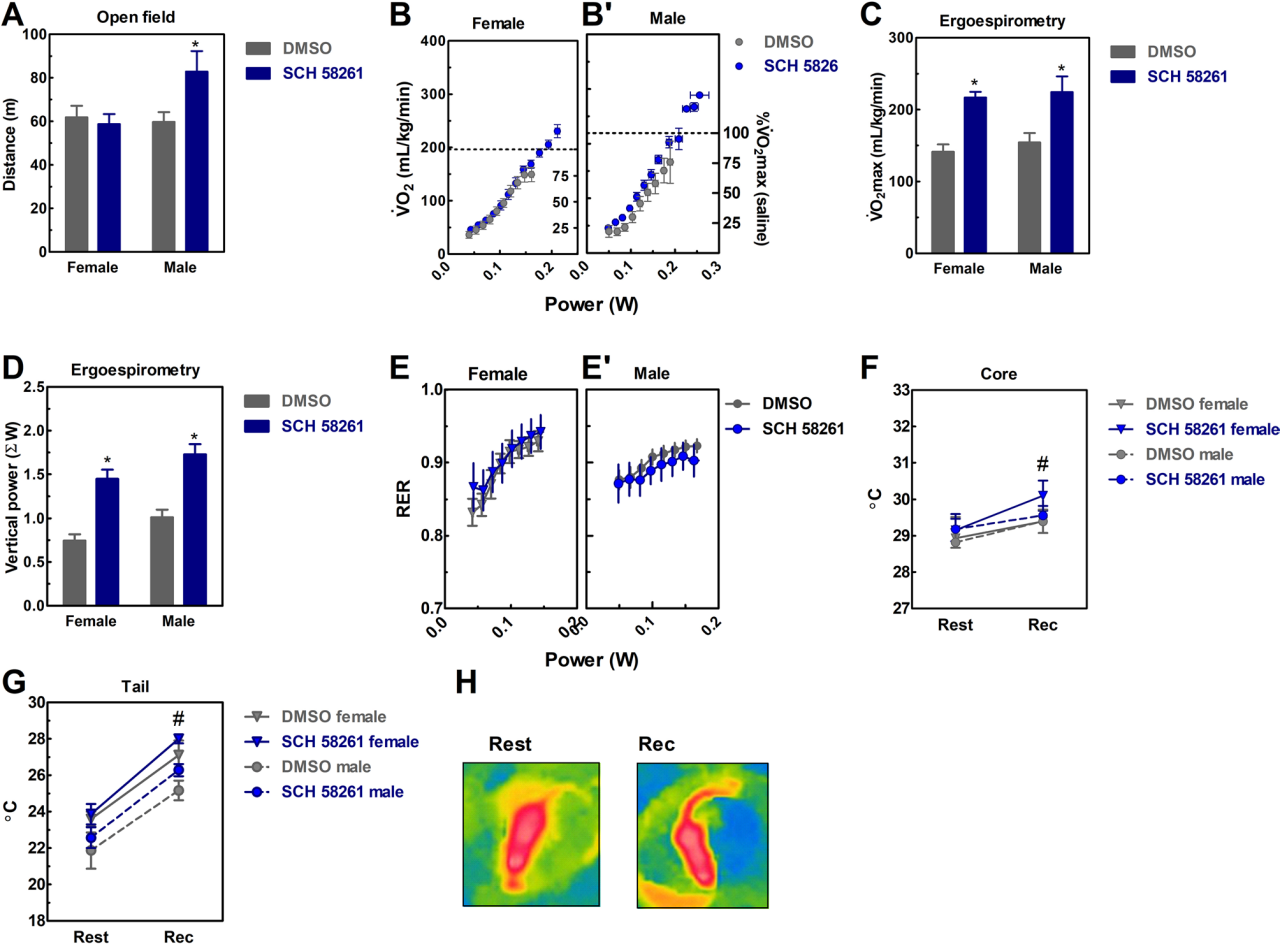
Effects of SCH 58261 (1 mg/kg, i.p.) on locomotion (**A**), ergospirometry (**B**–**E**), and thermoregulation (**F**–**H**) of wild type male and female mice. (**A**) SCH 58261 was psychostimulant only in males. (**B**) The dotted line represents the V̇O_2_max of the DMSO group. Ergospirometry increased V̇O_2_ (**B**), running power (**B**), and metabolic rate (**E**) until the animals reached fatigue. SCH 58261 was ergogenic in both sexes, as it increased V̇O_2_max (**C**) and running power (**D**). The animals presented exercise-induced core and tail hyperthermia (**H**), which was not 58261 modified by SCH (**F**–**G**). Sex was a significant factor in decreasing maximum responses to V̇O_2_ (**C**) and running power (**D**), and increasing core and tail temperature in females. Data are presented as mean ± SEM. N = 8–9 animals/group for 12 independent experiments. *P < 0.05 *vs.* DMSO (Two-way ANOVA followed by Newman-Keuls post hoc test). # *P* < 0.05 *vs.* rest (Repeated measures ANOVA followed by Bonferroni post hoc test). *DMSO* dimethyl sulfoxide. *Rec* recovery. *RER* Respiratory Exchange Ratio. *V̇O*_*2*_ oxygen consumption.

An infrared (IR) camera (FLiR C2, emissivity 0.95, FLiR Systems) placed overtop (25 cm height) of a plastic tube (25 cm diameter) was used to acquire a static dorsal thermal image^40,49,50^. IR images were taken immediately before and after exercise tests, namely at resting and recovery (Fig. 1H), respectively. IR images were analyzed with FLiR Tools software (Flir, Boston).

### Statistics

Data are presented as mean ± SEM in graphs built using the GraphPad Prism version 5.00 for Windows, GraphPad Software, San Diego California USA, www.graphpad.com.

Statistical analyzes were performed according to an intention-to-treat principle using StatSoft, Inc. (2007). STATISTICA (data analysis software system), version 8.0. www.statsoft.com. ANOVA two-way was used to evaluate open field, V̇O_2_max, running power, and resting and recovery temperature, followed by Newman-Keuls post hoc test. The evolution of submaximal V̇O_2_, running power, respiratory exchange ratio (RER), and heating were evaluated by ANOVA for repeated measures followed by Bonferroni post hoc test. The differences were considered significant when *P* < 0.05.

Effect sizes (Cohen's partial eta-square η^2^) were calculated for between-group changes in mean differences for V̇O_2_max, running power, and temperature, where a Cohen's η^2^ was used for ANOVA, defined as 0.01 small, 0.09 medium, and 0.25 large.

## Results

### SCH 58261: pharmacological inactivation of A_2A_R is ergogenic

SCH 58261 was psychostimulant for males, but not for females, since SCH 58261 only increased male locomotion in the open field (F_1,39_ = 4.5, η^2^ = 0.1, β = 0.54, 95% CI 58.8–72.1, *P* < 0.05, Fig. 1A).

The running power of females (F_7,77_ = 221, *P* < 0.05, Fig. 1B) and males (F_7,84_ = 183, *P* < 0.05, Fig. 1B') increased at each stage of the exercise test. Submaximal V̇O_2_ also increased to the maximum (V̇O_2_max, dotted line) of females (F_8,77_ = 168, *P* < 0.05, Fig. 1B) and males (F_7,84_ = 14.3, *P* < 0.05, Fig. 1B'). Female (F_8,70_ = 180, *P* < 0.05, Fig.S2A) and male (F_8,70_ = 164, *P* < 0.05, Fig.S2B) submaximal V̇CO_2_ kinetics was similar to V̇O_2_. SCH 58261 had no effect on these submaximal values.

We demonstrated for the first time that SCH 58261 is ergogenic since SCH 58261 increased V̇O_2_max (F_1,36_ = 27.7, η^2^ = 0.44, β = 0.99, 95% CI 0.16–0.2, *P* < 0.5, Fig. 1C) and running power (F_1,35_ = 55, η^2^ = 0.61, β = 1.0, 95% CI 1.0–1.3, *P* < 0.05, Fig. 1D) in both sexes.

SCH 58261 had no effect on increasing RER of females (F_7,70_ = 6.9, *P* < 0.5, η^2^ = 0.43, β = 0.99, Fig. 1E) and males (F_7,84_ = 9.4, η^2^ = 0.57, β = 0.99, *P* < 0.5, Fig. 1E’). Exercise test raised the animals' core (F_1,26_ = 5.5, η^2^ = 0.17, β = 0.62, 95% CI 28.7–29.39, *P* < 0.05, Fig. 1F) and tail temperature (F_1,22_ = 81, η^2^ = 0.78, β = 0.99, 95% CI 24.2–25.6, *P* < 0.05, Fig. 1G), with no effect of SCH 58261. Figure 1 shows the heating of the mouse's tail in post-exercise recovery (rec) in relation to rest. Three females at estrus (Fig.S3C) were excluded from temperature experiments due to large exercise-induced tail hyperthermia^40^. The previous results refer to females in diestrus (Fig.S3A), proestrus (Fig.S3B), and metestrus (Fig.S3D).

### Caffeine is not ergogenic in global A_2A_R knockouts

**Figure 2:**
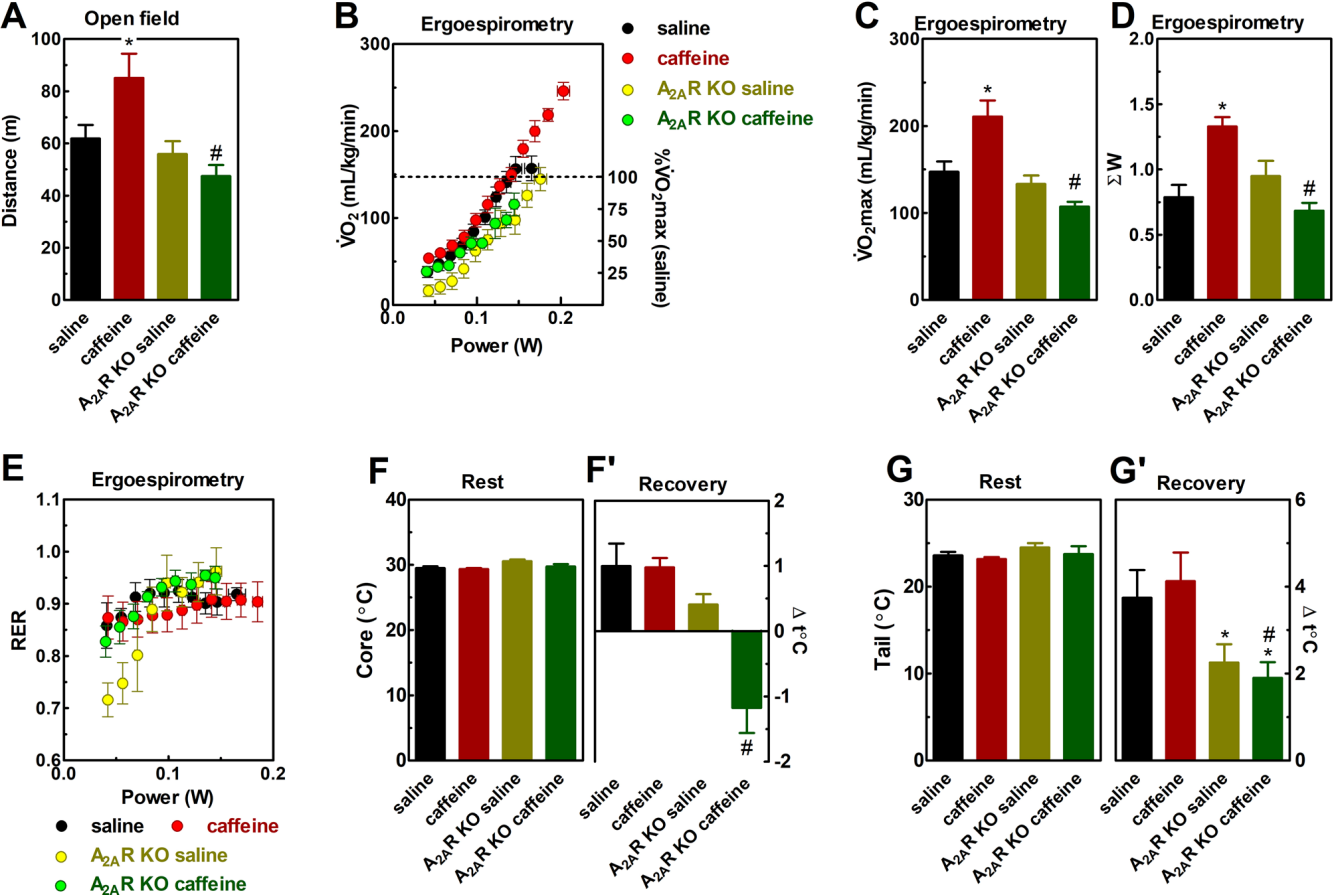
Effects of caffeine (15 mg/kg, i.p.) in wild type and global A_2A_R KO mice on locomotion (**A**), ergospirometry (**B**–**E**), and thermoregulation (**F**–**G**) of female mice. (**A**) Caffeine displayed a psychostimulant effect in the open field in wild type mice, but not in A_2A_R KO mice. Ergospirometry increased V̇O_2_ (**B**), running power (**B**), and metabolic rate (**E**) until the animals reached fatigue. The dotted line represents the V̇O_2_max of the wild type-saline group (**B**). Caffeine increased V̇O_2_max (**C**) and running power (**D**) of wild type, but not A_2A_R KO mice. Exercise test induced hyperthermia, which was not affected by caffeine in wild type mice, whereas caffeine caused a hypothermic response in A_2A_R KO mice (**F** and **G**). Genotype was a significant factor for V̇O_2_max (**C**), running power (**D**), resting (**F**), and recovery (**F**' and **G**') temperatures. Data are described as mean ± SEM. N = 8–9 animals/group for 12 independent experiments. *P < 0.05 *vs.* saline and # *P* < 0.05 *vs.* caffeine (Two-way ANOVA followed by Newman-Keuls post hoc test). *A*_*2A*_*R*—adenosine A_2A_ receptor. *KO*—knockout. *Rec* recovery. *RER* Respiratory Exchange Ratio. *V̇O*_*2*_ oxygen consumption.

Caffeine was psychostimulant based on its ability to increase locomotion in the wild type mice (F_1,36_ = 5.8, η^2^ = 0.13, β = 0.64, 95% CI 55.9–71.6, *P* < 0.05, Fig. 2A), an effect not seen in A_2A_R KO mice.

Figure 2B shows the progressive increase in submaximal V̇O_2_ (F_7,196_ = 255, *P* < 0.05), V̇CO_2_ (F_7,196_ = 189, *P* < 0.05, Fig.S2C) and running power (F_7,210_ = 6,243, *P* < 0.05) at speeds 35–50 cm/s, with less V̇O_2_ for A_2A_R KO mice. Caffeine was ergogenic but only in mice expressing A_2A_R. Caffeine improved V̇O_2_max (F_1,33_ = 12.6, η^2^ = 0.28, β = 0.93, 95 CI 0.12–0.17, *P* < 0.05, Fig. 2C) and running power (F_1,32_ = 22.3, η^2^ = 0.4, β = 0.99, 95% CI 0.84–1.09, *P* < 0.05, Fig. 2D) of wild type mice. The increase in critical power was 43.1 ± 7.1% concerning controls. A_2A_R KO did not display the ergogenic effects of caffeine.

Caffeine slowed the progression of RER in the wild type mice (F_7,133_ = 3.5, η^2^ = 0.15, β = 0.96, *P* < 0.05, Fig. 2E). Resting core (Fig. 2F) and tail (Fig. 2G) temperatures were similar between groups. Exercise increased the core (F_1,24_ = 0.16, η^2^ = 0.99, β = 0.99, 95% CI 29.5–30.2, *P* < 0.05, Fig. 2F’) and tail (F_1,25_ = 82, η^2^ = 0.73, β = 0.99, 95% CI 26.2–27.6, *P* < 0.05, Fig. 2G’) temperature of wild type animals. Caffeine did not change the exercise-induced core and tail heating, which was lower in the A_2A_R KO mice. Core temperature even dropped in caffeine-treated A_2A_R KO mice, as expected from the participation of A_1_R, also targeted by caffeine, on the control of body temperature^51^.

### Knocking out neuronal A_2A_R abrogates the ergogenic effects of caffeine

**Figure 3:**
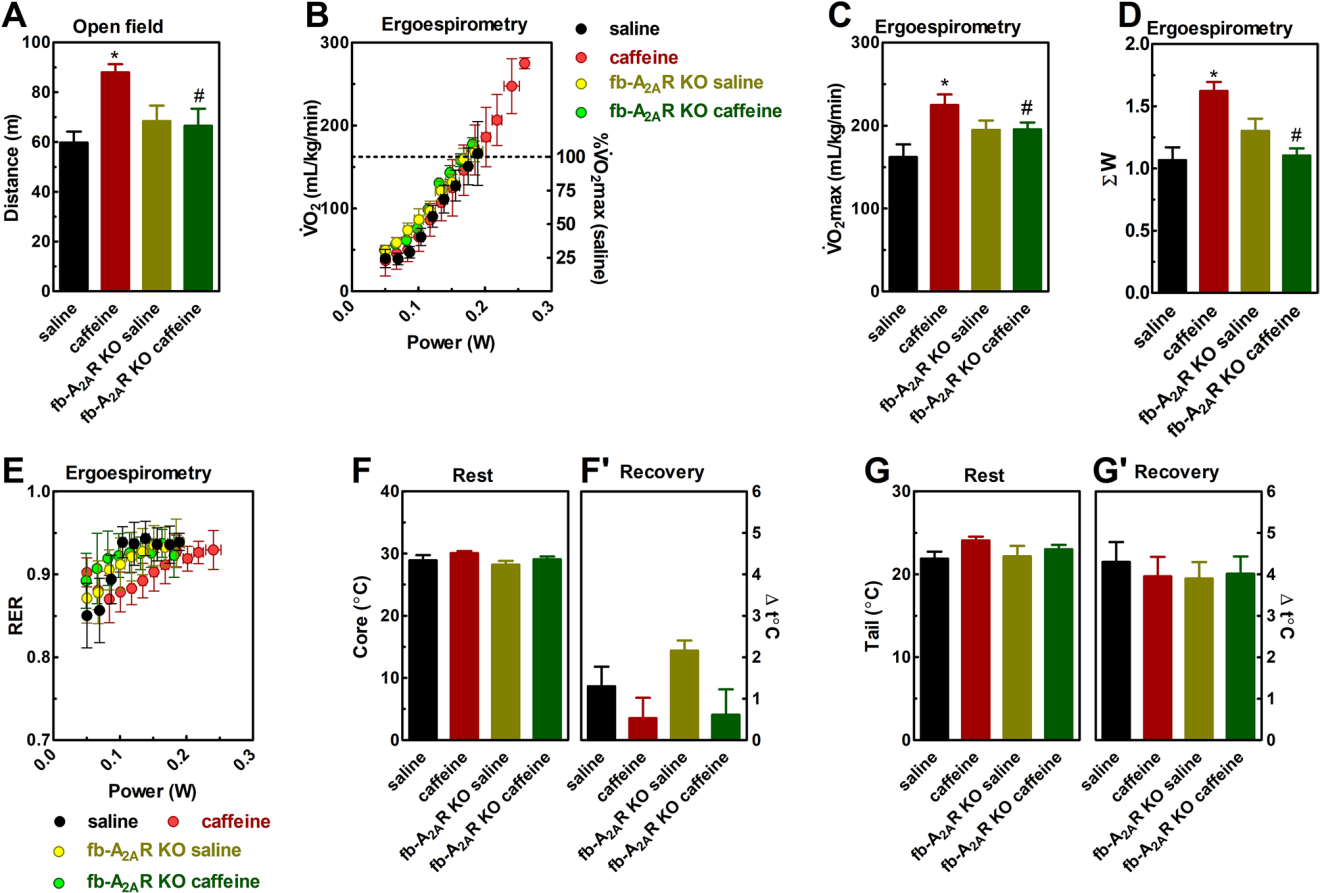
Effects of caffeine (15 mg/kg, i.p.) in wild type and forebrain A_2A_R KO mice on motor behavior (**A**), ergospirometry (**B**–**E**), and thermoregulation (**F**, **G**) of male mice. (**A**) Caffeine displayed a psychostimulant effect in the open field in wild type mice, but not in forebrain-A_2A_R KO mice. Ergospirometry increased V̇O_2_ (**B**), running power (**B**), and metabolic rate (**E**) until the animals reached fatigue. The dotted line represents the V̇O_2_max of the wild type-saline group (**B**). Caffeine increased V̇O_2_max (**C**) and running power (**D**) of wild type, but not forebrain A_2A_R KO mice. Resting core (**F**) and tail (**G**) temperature and tail heating (**G**') were similar between groups. Data are presented as mean ± SEM. N = 8–9 animals/group for 12 independent experiments. *P < 0.05 *vs.* saline and ^#^*P* < 0.05 *vs.* caffeine (Two-way ANOVA followed by Newman-Keuls post hoc test). *A*_*2A*_*R* adenosine A_2A_ receptor, *fb* forebrain, *KO* knockout, *Rec* recovery, *RER* respiratory exchange ratio. V̇O_2_ oxygen consumption.

The psychostimulant effect of caffeine was operated by A_2A_R since caffeine increased the locomotion of wild type males in the open field (F_1,34_ = 8.6, η^2^ = 0.11, β = 0.55, 95% CI 65.5–78.3, *P* < 0.05, Fig. 3A), but not in fb-A_2A_R KO mice.

Submaximal V̇O_2_ (F_7,147_ = 329, *P* < 0.05, Fig. 3B) and V̇CO_2_ (F_7,154_ = 359, *P* < 0.05, Fig.S2D) increased during the exercise test without caffeine and genotype effects. Caffeine was ergogenic but only in mice expressing neuronal A_2A_R. Caffeine increased V̇O_2_max (F_1,31_ = 5.7, η^2^ = 0.16, β = 0.64, 95% CI 0.17–0.21, *P* < 0.05, Fig. 3C) and running power (F_1,29_ = 4.4, η^2^ = 0.13, β = 0.98, 95% CI 1.16–1.39, *P* < 0.05, Fig. 3D) of wild type animals. The increase in critical power was 31.9 ± 4.7% concerning controls. Most importantly, caffeine was not ergogenic in fb-A_2A_R KO mice.

The increase in RER during the exercise test was lower in animals treated with caffeine (F_7,119_ = 3.6, η^2^ = 0.17, β = 0.97, *P* < 0.05, Fig. 3E), wild type, and fb-A_2A_R KO. Resting and recovery core temperatures were similar in all groups (Figs. 3F and Fig. 3G). The exercise test did not change the core temperature (Fig. 3F’). However, exercise heated the mice's tail in a similar way between groups (F_1,22_ = 102, η^2^ = 0.69, β = 0.99, 95% CI 24.9–26.4, *P* < 0.05, Fig. 3G').

## Discussion

### Neuronal A_2A_R antagonism is ergogenic

Caffeine increases exercise performance in rodents^16,17,19,26,40^ and humans^1,4,8–15,24,28,51,52^. Our results show the key role of A_2A_R in the ergogenic effects of caffeine using pharmacological and genetic tools. Thus, the potent and selective A_2A_R antagonist SCH 58261 displayed an ergogenic effect similar to that of caffeine, and the ergogenic effect of caffeine was abrogated in A_2A_R KO mice.

SCH 58261 and caffeine improved V̇O_2_max, running and critical power of wild type mice. These results are in line with the improved running time observed in caffeine-treated rats^16,26,53^ and mice^19^. Further evidence for the ergogenic effect of caffeine is based on its ability to increase muscle power and endurance output in rodents^54–58^. For the first time, we demonstrated that the selective antagonism of A_2A_R is ergogenic. Also, for the first time, we demonstrated that the genetic inactivation of A_2A_R impaired the ergogenic effects of caffeine. Tissue-specific A_2A_R KO selectively in forebrain neurons further allowed showing that these ergogenic effects of caffeine are due to the antagonism of A_2A_R in forebrain neurons. Thus, we suggest that caffeine decreases central fatigue during exercise. Moreover, caffeine decreased RER in the submaximal stages of the exercise test, an effect also abrogated in A_2A_R KO mice. However, exercise-induced core and tail hyperthermia were similar among animals treated with SCH 58261 or caffeine, except for A_2A_R KO mice, suggesting possible A_1_R-A_2A_R-mediated interactions^56,57^ in the temperature control^51^.

### Selective A_2A_R antagonism is psychostimulant in males, not females

We assessed the baseline motor behavior due to the motor nature of the running test, without any motor impairment found related to the different genotypes and treatments. Thus, the observed differences were not due to impaired animals' motor behavior. We also assessed the psychostimulant effects of caffeine and SCH 58261^34^. Notably, the effects of caffeine were abrogated in A_2A_R KO mice, and SCH 58261 did not modify locomotion in female mice. These results corroborate the robust evidence showing the psychostimulant effects of caffeine in male rodents^58^. However, little is known about the role of sexual dimorphism in adenosine signaling^59–63^. The absence of a psychostimulating effect of SCH 58261 in females is on step ahead, in notable agreement with the reported ability of the anxiolytic effect of SCH 58261 in males^59–61^ but not in females^60^. However, these differences did not disturb the ergogenic effects of SCH 58261 on females. Future studies will better understand sex differences in adenosine signaling, which was not the aim of this study.

### The neuropharmacology of the ergogenic effects of SCH 58261 and caffeine

Adenosine is a potent purine that modulates CNS signaling and functions from its main A_1_R and A_2A_R^21,23,29,62^. Here, caffeine (nonspecific A_1_R and A_2A_R antagonist) and SCH 58261 (selective A_2A_R antagonist) similarly increased the V̇O_2_max, running power, and critical power of exercising male and female mice. Most importantly, these ergogenic effects were abrogated by the selective deletion of A_2A_R in forebrain neurons, which indicates the key role of CNS A_2A_R as an ergogenic mechanism. The basal nuclei, namely the striatum, is the brain region with the highest density of A_2A_R^34,35,37,63^, which prompts the hypothesis that the A_2A_R antagonism in the basal ganglia might mediate the ergogenic effect of SCH 58261. In resting and running rodents, caffeine intake can result in a concentration of caffeine of 50 µM in the brain^19,64^. This concentration is close to the EC_50_ of caffeine (40 µM) to antagonize A_1_R and A_2A_R in the CNS^23^. Since caffeine was not ergogenic in fb-A_2A_R KO mice, it is concluded that forebrain A_2A_R signal the ergogenic effects of caffeine. This provides a direct demonstration of the involvement of neuronal A_2A_R in the ergogenic effects of caffeine, as suggested by two previous reports showing that NECA prevented the ergogenic effects of caffeine in rats^16^ and, conversely, that systemic caffeine reversed the poor running performance of NECA-treated rats^24^. Although nonselective, the pharmacological use of NECA demonstrated that adenosine receptors are crucial for the ergogenic effects of caffeine^16,26,53^. We now identified A_2A_R, specifically located in forebrain neurons, as responsible for this ergogenicity of caffeine.

The neurological effects of caffeine highlight its action on the CNS. Caffeine decreases the rate of perceived exertion^4–6,9,65^, pain^4,6,64,66,67^, central and mental fatigue during exercise^24,28,68,69^, indicating that caffeine attenuates mental fatigue during exercise. Caffeine also improves performance expectations^70^^,^ cognitive and executive functions^65,71–73^, and vigor^74^ in exercising subjects. The contribution of the CNS-mediated effects on exercise-induced fatigue conceptualizes central fatigue^24,28,51,52,68,75^. Caffeine reduces saccadic eyes fatigue^24,28^,also, the cortical silence of fatigued ankle muscles^51,52^. Moreover, caffeine increases spinal excitability^76^ and cortical motor area potentiation^68^ after exhausting exercise.

### Caffeine decreases RER in mice expressing peripheral A_2A_R

Caffeine decreases the RER in submaximal exercise in humans^1,77,78^ and rats^79^. For the first time, we provide evidence that this metabolic effect involves a modification of the A_2A_R function. In the past, the inhibition of phosphodiesterase and increased intracellular calcium mobilization^2,7,8,80^ were the proposed mechanisms. However, these proposals are inconsistent with pharmacological data: caffeine has an EC_50_ for adenosine antagonism of 40 µM, 1,000 mM for phosphodiesterase inhibition, and 3,000 mM for Ca^2+^-triggered muscle contraction^23^. Higher caffeine concentrations cause toxicity (above 200 µM) and lethality (above 500 µM)^23^. Thus, biological effects for caffeine must be in the range below 100 µM. We have previously shown that caffeine reaches a plasma peak of 10 µM after caffeine intake (6 mg/kg) in running mice^19^. The metabolic effects of caffeine during exercise are currently associated with increased activity of the autonomic nervous system (ANS)^1,19,77,78^, including high blood adrenaline and lactate levels, tachycardia and increased blood pressure. However, we must recognize the limitations of lung-based RER measures and their effects on metabolism, due to the possible artifacts such as hyperventilation and disturbances in the acid–base balance.

### Adenosine receptors are crucial for exercise-induced hyperthermia

The temperature changes observed were dependent on sex and genotype. The exercise test improved V̇O_2_, an index of heat production^26^^,^ but the core temperature increased only in females. The tail temperature, an index of heat loss^26^^,^ increased in both sexes. These results are in line with previous results from our group^40^. Caffeine and SCH 58261 did not modify these thermal responses to the exercise test. In males, tail heating of fb-A_2A_R KO mice was also similar to that of the wild type mice. However, the thermal response of the global A_2A_R KO females was different, indicating a peripheral role of these receptors, known to regulate body temperature^51^.

NECA (nonspecific A_1_R and A_2A_R agonist) causes core hypothermia in rats and rabbits^26,76^, an effect inhibited by 8-phenyltheophylline (a potent and selective antagonist for A_1_R and A_2A_R that crosses the blood–brain barrier, BBB)^76^^,^ but unaffected by 8-(p-sulfophenyl)theophylline (another potent adenosine receptor antagonist with little BBB penetration)^76^^,^ indicating a centrally-mediated effect. In the case of A_2A_R, its role in regulating body temperature is controversial^81^. The selective A_2A_R agonist 2-p-(2-carboxyethyl)-phenethylamino-5′-N-ethylcarboxamidoadenosine-hydrochloride (CGS 21680) induces hyperthermia in rats^82^ and mice^83^. We now show that SCH 58261 does not change resting and recovery temperature. This evidence suggests that the peripheral activation of A_2A_R can induce hypothermia in rodents, but this mechanism does not seem to be physiologically engaged as gauged by the lack of effects of the pharmacological or genetic blockade of A_2A_R. The previous data are from animals at rest—animals with CNS A_2A_R deletion present normal hyperthermia response during exercise. However, global A_2A_R KO displays a decreased response, even hypothermia, when treated with caffeine. Thus, A_2A_R seems to undergo a gain of function in the periphery during exercise. This data reinforces the well-known role of A_1_R in hypothermia^81^. Circulating adenosine levels increase during exercise^30,31^, and global A_2A_R KO imbalance appears to increase the A_1_R role, signaling hypothermia even after exercise. These results are limited to the use of infrared temperature, as we have not measured rectal temperature due to interference (vaginal manipulation) in the evaluation of the estrous cycle of females. Also, we kept the same methodology in males.

### Conclusion

In summary, we have now demonstrated that A_2A_R antagonism is a mechanism of action for ergogenicity, as SCH 58261 was ergogenic. Furthermore, we showed that the antagonism of forebrain A_2A_R was the mechanism underlying the ergogenic effect of caffeine since caffeine was not ergogenic in fb-A_2A_R KO. The use of selective A_2A_R KO in forebrain neurons further reinforces the ergogenic role of caffeine in decreasing central fatigue, with possible involvement of decreased perceived exertion, pain, and mental fatigue in humans. Despite methodological limitations, our data further suggest that caffeine modified the exercising metabolism in an A_2A_R-dependent manner and that A_2A_R is essential for exercise thermoregulation.

## Supplementary Information

Supplementary Figures.

## Data Availability

The datasets generated and analyzed during the current study are available from the corresponding author on reasonable request.
