# Supplementary material for: Neuronal adenosine A2A receptors signal ergogenic effects of caffeine
Source: Sci Rep. 2020 Aug 7;10:13414. doi: 10.1038/s41598-020-69660-1 (PMC7415152; doi:10.1038/s41598-020-69660-1)
Supplement: Supplementary file 1 — Supplementary Figures. [file 41598_2020_69660_MOESM1_ESM.docx]

# **Supplementary figures**

# **Neuronal adenosine A_2A_ receptors signal ergogenic effects of caffeine**

Aderbal S Aguiar Jr^1,2*^, Ana Elisa Speck^1,2^, Paula M. Canas^1^, Rodrigo A. Cunha^1,3^

^1^CNC-Center for Neuroscience and Cell Biology, University of Coimbra, 3004-504 Coimbra, Portugal; ^2^Biology of Exercise Lab, Department of Health Sciences, UFSC-Federal University of Santa Catarina, Araranguá, SC, Brazil 88905-120; ^3^FMUC – Faculty of Medicine, University of Coimbra, 3004-504 Coimbra, Portugal

* Corresponding author: Prof. Aderbal S Aguiar Jr, Universidade Federal de Santa Catarina – UFSC, Departamento de Ciências da Saúde, 88905-120, Araranguá – SC, Brazil. Tel.: +55 (48) 37214743. E-mail: [aderbal.aguiar@ufsc.br](mailto:aderbal.aguiar@ufsc.br)


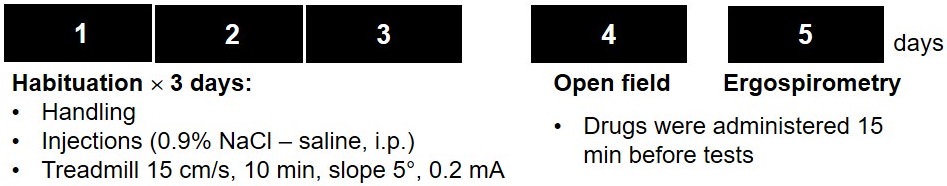


**Fig.S1** – Experimental design.


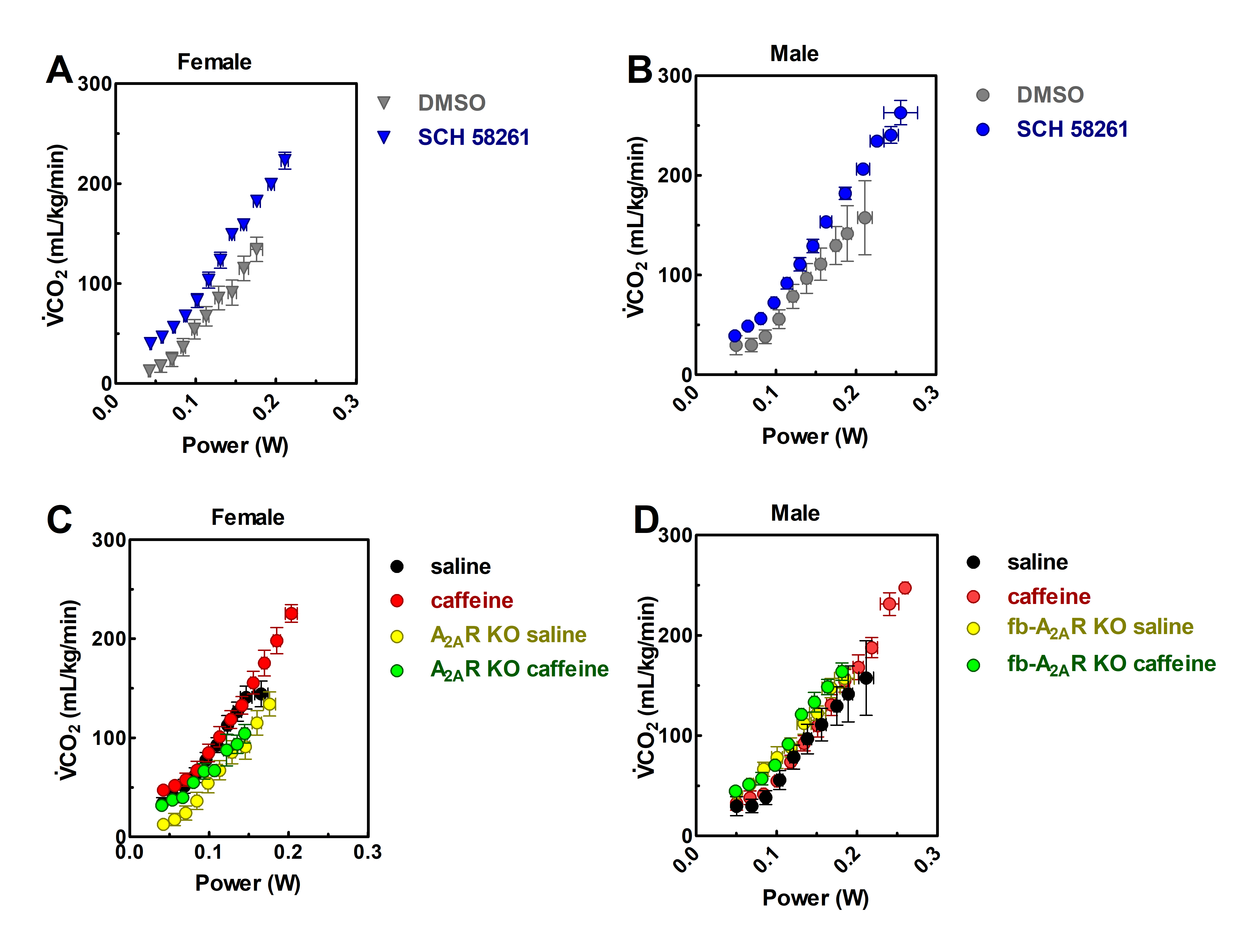


**Fig.S2** – Effects of SCH 58261 (1 mg/kg, i.p.) and caffeine (15 mg/kg, i.p) on submaximal V̇O_2_ in wild type, global A_2A_R KO and forebrain A_2A_R KO mice during an incremental exercise test. DMSO – Dimethyl sulfoxide. KO – Knockout. V̇CO_2_ – Carbon dioxide production.


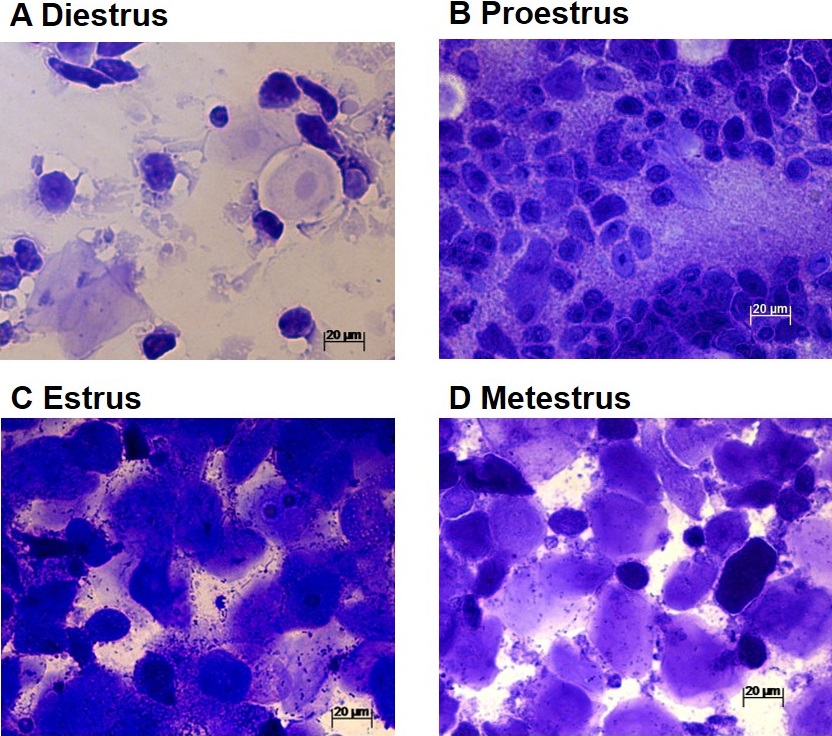


**Fig.S3** – Estrous cycle.
